# Prognostic Factors for Venous Thromboembolism in Patients with Solid Tumours on Systemic Therapy: A Systematic Review

**DOI:** 10.1055/a-1642-4572

**Published:** 2021-09-10

**Authors:** Sandra Lee, Anika Shenoy, Daniel Shi, Mootaz Husien, Pablo E. Serrano, Sameer Parpia

**Affiliations:** 1 Department of Surgery, McMaster University, Hamilton, Ontario, Canada; 2 Hamilton Health Sciences, Hamilton, Ontario, Canada; 3 Department of Medicine, Queen's University, Kingston, Ontario, Canada; 4 University of Waterloo, Waterloo, Ontario, Canada; 5 Department of Oncology, McMaster University, Hamilton, Ontario, Canada; 6 Department of Health Research Methods, Evidence, and Impact, McMaster University, Hamilton, Ontario, Canada

**Keywords:** venous thromboembolism, chemotherapy, neoplasms, prognosis

## Abstract

**Background** Patients undergoing systemic cancer therapy are susceptible to developing venous thromboembolism (VTE). The most pertinent prognostic factors for VTE remain unclear. This systematic review aims to summarize prognostic factors associated with VTE in this population.

**Methods** MEDLINE, Embase, and CENTRAL databases were searched for observational or randomized studies that used multivariable analysis adjusted for tumor type and/or metastatic disease to model the risk of VTE. Adjusted effect estimates for each prognostic factor were collected for all of the included studies. Risk of bias was assessed using the Quality in Prognostic Factor Studies (QUIPS) tool.

**Results** From 5,988 search results, 15 eligible studies and 42 prognostic factors were identified. A total of 8,554 patients of whom 456 (5.33%) developed VTE were included. Fourteen studies had a high risk of bias and one study had a moderate risk. The most commonly reported prognostic factors include age, gender, tumor site, metastasis, performance status, and systemic therapy type. Poor performance status and the use of platinum-based chemotherapy compounds were associated with an increased risk of VTE across the majority of studies. The evidence to suggest that the other prognostic factors identified were associated with VTE development was inconclusive. Several individual studies identified novel biomarkers for VTE. Heterogeneity in statistical methods and prognostic factor definitions across studies precluded meta-analysis.

**Conclusion** Overall, many prognostic factors were identified; however, the evidence for association with development of VTE for most of the factors is inconclusive. Findings were limited by high heterogeneity and risk of bias in the included studies.

## Introduction

Venous thromboembolism (VTE) is a common complication among patients with cancer, particularly in those receiving systemic chemotherapy or other antineoplastic treatments. 1 2 The incidence of VTE ranges from 2% to 8% in this population and has been identified as a leading cause of death in these patients. 3 4 5 6 Certain factors have been identified that further exacerbate the risk of VTE. For instance, certain solid tumors are associated with the highest risk of VTE, namely, pancreatic, stomach, and lung cancers. 1 4 6 7 Additionally, the presence of metastatic disease has been identified as a significant prognostic factor, increasing the risk of VTE by 4-fold to 13-fold compared with earlier stages. 1 4

The identification of such prognostic factors allows patients to be stratified into risk groups and aid in decisions to administer thromboprophylaxis prior to cancer treatment. As such, a widely used and validated scoring model, the Khorana score, predicts VTE in patients receiving chemotherapy on the basis of five clinical and laboratory parameters. 7 These include tumor site, body mass index, hemoglobin levels, platelet count, and leukocyte count. 7 Nonetheless, identifying patients who are at a high risk for VTE remains a challenge. A recent meta-analysis looked at 6-month VTE incidence by Khorana risk category in 27,849 cancer patients. 8 This study found that most VTE events occurred in the low- and intermediate-risk groups (11.6%) rather than the high-risk group (11.0%). Of the patients who were diagnosed with VTE, 23.4% were classified as high-risk according to the Khorana score. 8 Newer studies have since identified additional prognostic factors, including predictive biomarkers, for VTE. For instance, Ay et al. observed that adding D-dimer and soluble P-selectin as variables to the Khorana score more accurately predicted VTE. 9 Additionally, certain types of chemotherapy regimens were found to significantly increase the risk of VTE. 10

Although many studies have investigated various clinical and pathological factors that predict VTE, these results have yet to be systematically examined. Therefore, this systematic review was conducted to identify and summarize the most pertinent prognostic factors associated with the development of VTE in patients with solid tumors undergoing systemic therapy.

## Methods

This systematic review was conducted and reported according to the Preferred Reporting for Systematic Reviews and Meta-Analyses (PRISMA) guidelines. 11 The protocol of this study was registered prior to study commencement in the Prospective Register of Systematic Reviews (CRD42020165501).

### Search Strategy

A comprehensive search of MEDLINE, Embase, and the Cochrane Central Register of Controlled Trials (CENTRAL) databases was conducted, obtaining records from database conception to August 2019 inclusive. Highly sensitive search strategies unique to each database were developed ( Supplementary Table S1 , 2 , 3 ). Subject headings and key words were used to form the strategy, which included terms such as venous thromboembolism, cancer, systemic therapy, and their synonyms. These phrases were combined with the methodology term ‘prognosis’. References of included studies were hand-searched to identify any additional relevant studies.

### Eligibility

Studies were eligible for inclusion if they reported a multivariable analysis to model the risk of VTE in patients with solid tumors undergoing chemotherapy or other types of systemic therapy. VTE was defined as symptomatic and incidental pulmonary embolism and deep vein thrombosis. Only patients with solid tumors, including lymphomas, were included in this review. Primary brain tumors and leukemias were excluded due to differences in treatment course and staging compared with other solid tumors. Additionally, patients who received thromboprophylaxis or underwent surgery during the treatment or follow-up period were excluded. Studies of any study design available in English were included.

### Data Abstraction

Titles and abstracts were screened by two independent reviewers using pre-defined inclusion and exclusion criteria. Full-text review was also conducted by two independent reviewers, with the most important reason for exclusion documented during this stage. Conflicts at each stage were resolved through discussion and consensus. A third reviewer was consulted if consensus could not be reached. Agreement between reviewers was calculated using Cohen's kappa (k) for each step of the screening process.

Data was extracted by two independent reviewers using a standardized, pilot-tested form designed *a priori* . Study characteristics, patient demographics, tumor characteristics, treatment types, VTE incidence, and all prognostic factors reported by each study were recorded. For studies that performed multivariable analysis on a subset of patients, only the subset was considered for this review. Adjusted effect estimates for each prognostic factor were drawn, which were determined using odds ratios (ORs), hazard ratios (HRs), and sub-distributed hazard ratios (SHRs). For studies that did not report full multivariable analysis results, corresponding authors were contacted for this information. To avoid double-counting patients that were included in more than one study, corresponding authors of studies conducted by the same research group were contacted for clarification around overlapping cohorts.

### Selection of Prognostic Factors

The purpose of this review was to identify the most pertinent prognostic factors for VTE, thus, only studies with adjusted effect estimates as modeled in multivariable analyses were included. To further standardize the effect estimates collected for each prognostic factor, only studies that adjusted for at least one of the two factors (i.e., tumor site and/or metastasis) were included. These factors were selected for their strong association with VTE development according to previous literature. 1 4 12 Studies with effect estimates that did not adjust for metastasis and/or tumor type were excluded from this review, as well as studies that did not report a measure of association for prognostic variables.

### Statistical Analysis

Baseline patient characteristics were summarized using descriptive statistics based on the available data in the included studies. If multiple studies reported on overlapping patient cohorts, the study with the largest sample size from each research group was prioritized when calculating baseline characteristics.

Meta-analysis of prognostic factors using the DerSimonian and Laird random effects approach was considered if a prognostic factor was reported in four or more studies using the same effect measure.

### Risk of Bias Assessment

The quality of each study was assessed using the Quality in Prognostic Factor Studies (QUIPS) tool. 13 The QUIPS tool assigns the risk of bias as low, moderate, or high in six domains: study participation, study attrition, prognostic factor measurement, outcome measurement, study confounding, and statistical analysis and reporting. The domain with the highest risk of bias was used to determine the overall study quality.

## Results

### Study Characteristics

**Fig. 1:**
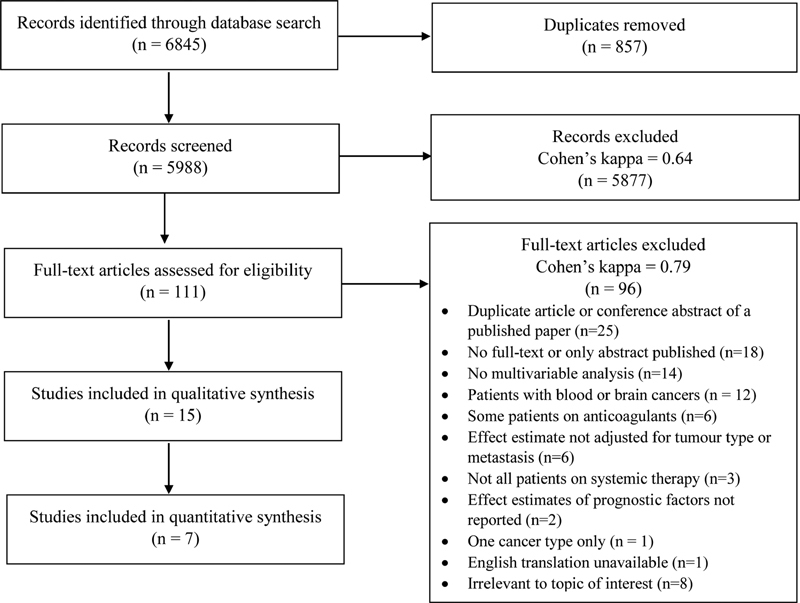
Flow diagram of study selection.

The search strategy identified 5,988 studies of which 111 underwent full-text review. Fifteen studies were eligible for final inclusion ( Fig. 1 ). A descriptive summary of the included studies can be found in Table 1 . There was substantial agreement for both title and abstract screening (k = 0.64) and full-text review (k = 0.79).

**Table 1:** Characteristics of included studies

Author and year	Study design	Country	Sample size	Median follow-up (months)
Abdel-Razeq 2018 23	Retrospective	Jordan	1677	>4 weeks, median not specified
Arpaia 2009 25	Prospective	Italy	124	6, median not specified
Di Nisio 2019 22	Retrospective	Netherlands	776	10.8 (IQR 5.2–12)
Ferroni (GFR) 2014 14 *	Retrospective	Italy	322	9.3
Ferroni (MPV) 2014 15 *	Prospective	Italy	589	8.5
Ferroni 2015 17 *	Retrospective	Italy	810 (380 in analysis)	9.2
Ferroni 2016 16 *	Retrospective	Italy	297	14
Gerotziafas 2017 27	Prospective	France, Lebanon, Jordan, Saudi Arabia, Kuwait, Syria	1023	6, median not specified
Khorana 2005 19 **	Prospective	United States	3003	2.4
Khorana 2008 7 **	Prospective	United States	4066 (2701 in analysis)	2.4 (range 0.2–12.0)
Roselli 2013 18 *	Prospective	Italy	505	11.2
Tafur 2015 24	Prospective	United States	241	Mean 10.4 (SD 3.2)
van Es 2017 20 ***	Prospective	Netherlands, Italy, Mexico, France	876	6, median not specified
van Es 2018 21 ***	Prospective	Netherlands, Italy, Mexico, France	648	5.9 (IQR 3.2–5.9)
Vergati 2013 26	Retrospective	Italy	486	12

Abbreviations: IQR, interquartile range; SD, standard deviation.

* Study conducted by same research group.

** Study conducted by same research group.

*** Study conducted by same research group.

The included studies were published between 2005 and 2019, with six of the 15 studies published in the past five years. Five studies were conducted in Italy by the same research group, 14 15 16 17 18 two studies in the United States by another research group, 7 19 and two studies with centres in the Netherlands, Italy, Mexico, and France were conducted by another research group. 20 21 For the remaining studies, one was conducted in the Netherlands, 22 one in Jordan, 23 one in the United States, 24 two in Italy, 25 26 and one study with centres across France, Lebanon, Jordan, Saudi Arabia, Kuwait, and Syria. 27 Nine studies were prospective cohorts, 7 15 18 19 20 21 24 25 27 and 6 studies were retrospective cohorts. 14 16 17 22 23 26

### Assessment of Quality

All studies were noted to have some degree of methodological flaws ( Supplementary Table S4 ). In the majority of studies, the process of identifying and selecting participant was unclear, resulting in a high risk of bias in study participation. There was moderate to high risk of bias for study attrition as the loss to follow-up rates and reasons were not explicitly described in most studies. Consideration of all prognostic factors, including variations in treatment, was incomplete in most studies, resulting in a moderate to high risk of bias for study confounding. Most studies did not provide sufficient detail describing the analysis models, such as how variables were defined in the analysis and the selection process for the variables that were included in the model, which led to a moderate to high risk of bias for statistical analysis and reporting.

### Patient Characteristics

Baseline characteristics of each included study are available in Table 2 . Considering only the study with the largest sample size when multiple studies reported on overlapping patient cohorts, this review includes a total of 8,554 patients of which 456 (5.33%) developed VTE. The median follow-up time in each study ranged from 2 to 14 months. The mean age ranged from 50 to 65 years old across studies. Of the included patients, the most common types of cancers that were reported were breast (27.9%), lung (20.5%), and colorectal (14.7%). Overall, 3,967 (46.4%) of patients had metastatic disease. Of the studies that reported baseline Khorana score risk category, 1,313 (30.1%) patients were at low risk (0 points), 2,538 (58.2%) at intermediate risk (1–2 points) and 510 (11.7%) at high risk (3+ points). Of the studies that reported baseline Eastern Cooperative Oncology Group (ECOG) performance status, 4,855 (82.7%) patients had a status of 0–1 points and 416 (7.1%) patients had a status of 2–4 points.

**Table 2:** Baseline patient characteristics

Author and year	Sample size	VTE, n (%)	Age (years) a	Female, n (%)	Metastatic, n (%)	Khorana risk category, n (%)		ECOG Status, n (%)	
Abdel-Razeq 2018	1677	110 (6.6)	50 (18–83)	578 (35)	857 (51)	Low	350 (21)	Not specified	
			Med (Range)			Intermediate	1075 (64)		
						High	252 (15)		
Arpaia 2009	124	11 (8.9)	64 (9)	61 (49)	57 (46)	Not specified		Not specified	
Di Nisio 2019	776	69 (8.9)	65 (11)	305 (39)	540 (7)	Low	172 (22)	0 to 1	736 (94.8)
						Intermediate	505 (65)	2 or more	37 (4.8)
						High	89 (12)	Missing	3 (0.4)
						Missing	9 (1)		
Ferroni (GFR)* 2014	322	25 (7.8)	61 (11)	143 (44)	172 (53)	Low	120 (37)	Not specified	
						Intermediate	177 (55)		
						High	25 (8)		
Ferroni (MPV)* 2014	589	40 (6.8)	62 (12)	300 (51)	201 (34)	Low	264 (45)	Not specified	
						Intermediate	289 (49)		
						High	36 (6)		
Ferroni* 2015	380	31 (8.2)	≤65 years, 193 (51); > 65 years, 187 (49)	172 (45)	169 (44)	Low	0	0 to 1	373 (98)
						Intermediate	380 (100)	2 or more	7 (2)
						High	0		
Ferroni* 2016	297	26 (8.8)	63 (10)	161 (54)	135 (45)	Low	171 (57)	0 to 1	296 (99)
						Intermediate	106 (36)	2 or more	1 (1)
						High	20 (7)		
Gerotziafas 2017	1023	88 (8.6)	55 (12)	821 (81)	405 (40)	Not specified		0 to 1	920 (90)
								2 or more	103 (10)
Khorana** 2005	3003	58 (2)	60	2005 (67)	1075 (36)	N/A		0 to 1	2717 (91)
								2 or more	272 (9)
Khorana** 2008	2701	60 (2.2)	<65 years, 1618 (60); ≥65 years, 1083 (40)	1819 (67)	997 (37)	N/A		0 to 1	2473 (92)
								2 or more	228 (8)
Roselli* 2013	505	35 (6.9)	60 (11)	265 (52)	282 (56)	Low	271 (54)	0 to 1	504 (99)
						Intermediate	205 (40)	2 or more	2 (1)
						High	29 (6)		
Tafur 2015	241	31 (12.9)	60 (53–68)	173 (72)	78 (32)	Low	55 (23)	0 to 1	221 (92)
			Med (IQR)			Intermediate	154 (64)	2 or more	20 (8)
						High	32 (13)		
van Es*** 2017	876	53 (6.1)	64 (11)	360 (41)	581 (66)	Low	265 (30)	Not specified	
						Intermediate	473 (54)		
						High	105 (12)		
						Missing	33 (4)		
van Es*** 2018	648	40 (6.2)	62 (11)	281 (43)	373 (58)	Not specified		0 to 1	599 (92)
								2 or more	49 (8)
Vergati 2013	486	33 (6.8)	61 (11)	262 (54)	251 (52)	Low	262 (54)	0 to 1	482 (99)
						Intermediate	196 (40)	2 or more	4 (1)
						High	28 (6)		

Abbreviations: ECOG, Eastern Cooperative Oncology Group; NSCLC, Non-small cell lung carcinoma.

a Age is reported as mean (standard deviation), unless indicated otherwise

* Study conducted by same research group

** Study conducted by same research group

*** Study conducted by same research group

## Prognostic Factors for VTE

From the included studies, a total of 42 unique prognostic factors for VTE were identified. Table 3 lists the classification and frequency of each prognostic factor explored in the included studies. The multivariable analysis results of each prognostic factor identified in this review are available in Supplementary Table S5 . Due to the heterogeneity in patient population, outcomes reported, definition of prognostic factors, and statistical methods used across studies, we opted against meta-analyzing the data and provide a narrative synthesis.

**Table 3:** Classification of prognostic factors and number of studies reporting on each

**Patient factors**
Age ( *n* = 8), gender ( *n* = 8), BMI ( *n* = 4), ECOG ( *n* = 8), Khorana score ( *n* = 7), previous VTE ( *n* = 1), vascular/lymphatic macroscopic compression ( *n* = 1), cardiovascular comorbidities ( *n* = 1)
	**Tumour factors**
Tumour site ( *n* = 9), metastasis ( *n* = 11), time since cancer diagnosis ( *n* = 1)
**Biomarkers**
Prechemotherapy hemoglobin ( *n* = 5), prechemotherapy white blood cell count ( *n* = 4), prechemotherapy platelet count ( *n* = 7), low protein C activity ( *n* = 1), high factor VIII activity ( *n* = 1), ThromboPath change ( *n* = 1), D-dimer levels ( *n* = 4), fibrin generation ( *n* = 1), eGFR ( *n* = 1), platelet/lymphocyte ratio ( *n* = 1), neutrophil/lymphocyte ratio ( *n* = 1)
	**Treatment Factors**
	Hospitalization ( *n* = 1), adjuvant chemotherapy ( *n* = 1), central venous catheter ( *n* = 2), gemcitabine ( *n* = 7), platinum ( *n* = 7), fluoropyrimidine ( *n* = 5), irinotecan ( *n* = 3), anthracycline ( *n* = 5), docetaxel ( *n* = 4), pemetrexed ( *n* = 3), bevacizumab ( *n* = 3), herceptin ( *n* = 3), anti-tyrosine kinase inhibitors ( *n* = 6), endocrine/anti-hormonal therapy ( *n* = 2), erythropoietin stimulating agents ( *n* = 4), prophylactic myeloid growth factors ( *n* = 4), corticosteroids ( *n* = 5)
**Genetic factors**	
VEGFA-1190G/A A/A polymorphism ( *n* = 1), VEGFA-1154G/A A/A polymorphism ( *n* = 1), VEGFA-634G/C C/C polymorphism ( *n* = 1)	

### Patient Factors

The most commonly reported patient-related prognostic factors were age, gender, and ECOG performance status. Age was analyzed in eight studies (or four patient cohorts). One retrospective cohort study found older age to be a predictor of VTE. 26 Sex was explored in eight studies (or four patient cohorts). Female was associated with an increased risk of VTE in one retrospective study, 23 however, the remaining studies found no evidence of association. Eight studies (or four patient cohorts) examined ECOG performance status. Five of these studies (or two patient cohorts) found poor ECOG status to be associated with a higher risk of VTE. 15 16 17 18 26

### Tumour Factors

Tumour site and the presence of metastasis were the most commonly investigated tumor-related factors. Nine studies assessed tumor site. Five studies examined the risk of VTE in patients with gastric and pancreatic tumors compared with breast, colorectal, and head-neck tumors. Of these five studies, two prospective studies (reporting on the same patient cohort) found an increased risk of VTE in patients with gastric and pancreatic tumors compared with the other sites. 7 19 One multi-institutional prospective study compared only gastric tumors to other sites and found an increased risk of VTE in patients with gastric tumors. 27 The evidence to suggest that lung, lymphoma, gynecologic, and genitourinary cancers grouped together were associated with a higher risk of VTE when compared with breast, colorectal and head-neck cancers in all three studies that made this comparison was inconclusive. 7 20 22 Presence of metastasis was investigated in eleven studies. Of the seven patient cohorts represented across these studies, one retrospective study found that metastasis increased the risk of VTE, 23 however, the remaining studies found no evidence of association.

### Biomarkers

Eleven biomarkers were assessed to predict VTE. Prechemotherapy platelet count was explored in seven studies representing five patient cohorts and was found to be associated with a higher risk of VTE in four of these studies (or three patient cohorts). 7 15 19 27 Prechemotherapy hemoglobin was explored in five studies representing four patient cohorts. One patient cohort found that low levels of hemoglobin or the use of erythropoietin stimulating agents increased the risk of VTE, 7 19 while the other studies found no evidence of such association. Several individual studies identified novel biomarkers that were associated with VTE. The Ferroni et al. and Roselli et al. study group observed that high platelet-lymphocyte ratio, impaired estimated glomerular filtration rate (eGFR), and decreased activated protein C function were associated with an increased risk of VTE. 14 17 18 Tafur et al. found low protein C levels to be a predictor of VTE, along with high factor VIII levels. 24

### Treatment Factors

Seventeen treatment-related factors for the development of VTE were identified. Gemcitabine and platinum-based compounds were the most commonly investigated treatment types. Across the seven studies (or four patient cohorts) exploring gemcitabine use, one study observed a higher risk of VTE in gemcitabine-treated patients compared with those who were administered other treatment regimens. 20 Seven studies representing four patient cohorts investigated platinum-based compounds. All four patient cohorts reported the use of platinum-based compounds to increase the risk of VTE compared with non-platinum-based treatments. 16 18 20 22 26 The most commonly investigated biological therapy was bevacizumab, which was reported in six studies representing two patient cohorts. One patient cohort found that bevacizumab was associated with a higher risk of VTE when compared with other treatments. 16 17 Seven studies reporting on four patient cohorts investigated the effect of supportive drug use during chemotherapy, including erythropoietin stimulating agents, prophylactic myeloid growth factors and corticosteroids. The evidence across these studies to suggest that use of these drugs was associated with VTE development was inconclusive.

### Genetic Factors

Ferroni et al. investigated genetic factors and observed that certain vascular endothelial growth factor (VEGFA) gene promoter variants, specifically, single nucleotide polymorphisms (SNPs) in these regions, were protective against VTE. 16 The frequencies of each VEGFA SNP genotype in the study sample were comparable to a group of healthy controls.

## Discussion

This systematic review identified pertinent prognostic factors in the literature for the development of VTE in patients with cancer undergoing systemic treatments. We identified the use of platinum-based chemotherapy compounds and poor performance status as important prognostic factors for VTE.

Currently, the most commonly recognized prognostic factors are those identified by Khorana et al. 7 A recent systematic review and meta-analysis assessed the effectiveness of thromboprophylaxis in patients at an intermediate to high risk of VTE according to the Khorana score. For these patients, both direct oral anticoagulants (DOACs) and low molecular weight heparin (LMWH) significantly decreased the incidence of VTE. 28 However, the administration of DOACs doubled the risk of major bleeding compared with the placebo group. Additionally, several trials in the review reported a higher risk of clinically relevant non-major bleeding in groups receiving DOAC or LMWH compared with placebo. 28 Thus, it is important to carefully consider the risks associated with anticoagulants against the benefits, and to appropriately identify patients who are at risk of developing VTE. One of the limitations of the Khorana score was that most patients used to derive the scoring model had a good performance status. 29 However, in more recent studies identified by this review, poor performance status (i.e., ECOG performance status ≥2) increased the risk of VTE and should be considered independently when assessing patients for VTE risk.

Given these limitations, modified versions of the Khorana score which take into consideration additional clinical and biomarker-based variables have been created, such as the Vienna CATS 9 and PROTECHT 30 scores. One multinational, prospective cohort study observed greater predictive power by these two scores compared with the Khorana score, mainly due to the addition of D-dimer levels and chemotherapy type as variables. 20 However, drawbacks of these scores include difficulty measuring D-dimer in clinical practice and an increased complexity of administering scores as more items are added. In the same study, the incidence of VTE was still appreciable in patients classified as low-risk by these scores, with 6-month rates of 5–6% in low-risk patients and 8–10% in high-risk patients. 20

Metastasis has previously been reported as a significant prognostic factor for VTE. 1 4 In the current review, however, the evidence to suggest that metastasis was associated with a higher risk of VTE was inconclusive. Chew et al. conducted one of the largest database studies of cancer-associated thrombosis to date and found that for all cancer types analyzed, metastatic disease increased the risk of VTE compared with localized disease. 4 However, the risk of VTE is exacerbated by the administration of certain anticancer treatments. 18 30 31 32 Thus, the effect of metastasis may have been lost when adjusted for treatment type in the included studies. Another possible explanation is the exclusion of patients with leukemia. Previous studies evaluating metastasis on the risk of VTE included patients with non-solid tumors. 1 31 Given the high incidence of VTE in patients with leukemia, 4 33 inclusion of these patients may have acted as a confounding factor. Additionally, metastasis did not have a consistent definition across individual studies, which may explain the lack of association with VTE. Some studies included locally advanced disease in their reference group (i.e., non-metastatic group), while some studies did not specify. Given that both locally advanced and distant cancer stages are independent predictors of VTE, 34 this may partly describe the low difference in VTE risk between metastatic and non-metastatic groups in the included studies.

Gemcitabine and platinum-containing chemotherapy were the most commonly reported treatment-related prognostic factors for VTE. Similar to previous studies, 35 36 37 we found that the use of platinum-based compounds was associated with an increased risk of VTE across the included studies. The association between the use of gemcitabine and VTE was less conclusive, despite previous studies suggesting that this treatment increased the risk of VTE, 37 38 which may be explained by adjustment factors and potential confounding.

There are several important limitations to consider. First, all of the included studies were assessed as having a moderate to high risk of bias. One of the main sources of bias was potential confounding. Although studies investigated the risk of chemotherapy types and supportive drugs, details such as dose and duration of these treatments were unavailable, which may have acted as sources of confounding. Other confounding variables may have been missed, as most studies did not provide an adequate explanation of how their multivariable models were built and how they selected which factors to include in their model. Additionally, there was poor reporting on the analysis models which acted as another major source of bias. Many studies did not describe how they defined certain prognostic factors in their multivariable analyses, nor how these statistical models were developed. Second, while some variables were prognostic in individual studies, we were not able to confirm their association with VTE in this review. This was due to large differences in the analysis of prognostic factors across studies, as our findings were based on the summary results of individual studies. Although we controlled for at least two types of adjustment factors (i.e., tumor type and/or metastasis), studies used a diverse set and number of adjustment factors in their analyses to estimate the effect of each prognostic factor. Moreover, prognostic factors had varied definitions and cut-offs across studies or lacked a clear explanation of how they were defined in the multivariable analysis altogether. Different summary measures were also used across studies. Due to these limitations, we were unable to perform meta-analysis for the prognostic factors that were identified. In this review, only adjusted risk estimates, with adjustment factors defined a priori, were collected. Although this partly standardized the risk estimates that were collected, a large number of studies were excluded on the basis of this criteria. Thus, risk factors that may be prognostic in excluded individual studies are not reported. Finally, many studies relied on a low number of VTE events or very few participants who possessed a prognostic factor, which may have overestimated the effect of some factors in the individual studies.

Overall, many prognostic factors were identified in this review, but the effect of these factors on VTE risk is inconclusive. These findings should be interpreted in light of the high risk of bias of included studies and heterogeneity across studies. There was an overall poor quality in reporting which is an area for improvement for future prognostic studies.

## References

[1] Blom J W Vanderschoot J P Oostindiër M J Osanto S van der Meer F J Rosendaal F R Incidence of venous thrombosis in a large cohort of 66,329 cancer patients: results of a record linkage study J Thromb Haemost 2006 4 03 529 535 10.1111/j.1538-7836.2006.01804.x 16460435

[2] Heit J A Silverstein M D Mohr D N Petterson T M O'Fallon W M Melton L J III Risk factors for deep vein thrombosis and pulmonary embolism: a population-based case-control study Arch Intern Med 2000 160 06 809 815 10.1001/archinte.160.6.809 10737280

[3] Sallah S Wan J Y Nguyen N P Venous thrombosis in patients with solid tumors: determination of frequency and characteristics Thromb Haemost 2002 87 04 575 579 12008937

[4] Chew H K Wun T Harvey D Zhou H White R H Incidence of venous thromboembolism and its effect on survival among patients with common cancers Arch Intern Med 2006 166 04 458 464 10.1001/archinte.166.4.458 16505267

[5] Khorana A A Francis C W Culakova E Kuderer N M Lyman G H Thromboembolism is a leading cause of death in cancer patients receiving outpatient chemotherapy J Thromb Haemost 2007 5 03 632 634 10.1111/j.1538-7836.2007.02374.x 17319909

[6] Lyman G H Eckert L Wang Y Wang H Cohen A Venous thromboembolism risk in patients with cancer receiving chemotherapy: a real-world analysis Oncologist 2013 18 12 1321 1329 10.1634/theoncologist.2013-0226 24212499 PMC3868427

[7] Khorana A A Kuderer N M Culakova E Lyman G H Francis C W Development and validation of a predictive model for chemotherapy-associated thrombosis Blood 2008 111 10 4902 4907 10.1182/blood-2007-10-116327 18216292 PMC2384124

[8] CAT-prediction collaborators Mulder F I Candeloro M Kamphuisen P W The Khorana score for prediction of venous thromboembolism in cancer patients: a systematic review and meta-analysis Haematologica 2019 104 06 1277 1287 10.3324/haematol.2018.209114 30606788 PMC6545838

[9] Ay C Dunkler D Marosi C Prediction of venous thromboembolism in cancer patients Blood 2010 116 24 5377 5382 10.1182/blood-2010-02-270116 20829374

[10] Barni S Labianca R Agnelli G Chemotherapy-associated thromboembolic risk in cancer outpatients and effect of nadroparin thromboprophylaxis: results of a retrospective analysis of the PROTECHT study J Transl Med 2011 9 179 10.1186/1479-5876-9-179 22013950 PMC3220644

[11] Moher D Liberati A Tetzlaff J Preferred reporting items for systematic reviews and meta-analyses: the PRISMA statement Ann Intern Med 2009 151 04 264 9, W64. 10.7326/0003-4819-151-4-200908180-00135 19622511

[12] Wun T White R H Epidemiology of cancer-related venous thromboembolism Best Pract Res Clin Haematol 2009 22 01 9 23 10.1016/j.beha.2008.12.001 19285269 PMC2702321

[13] Hayden J A van der Windt D A Cartwright J L Côté P Bombardier C Assessing bias in studies of prognostic factors Ann Intern Med 2013 158 04 280 286 10.7326/0003-4819-158-4-201302190-00009 23420236

[14] Ferroni P Guadagni F Laudisi A Estimated glomerular filtration rate is an easy predictor of venous thromboembolism in cancer patients undergoing platinum-based chemotherapy Oncologist 2014 19 05 562 567 10.1634/theoncologist.2013-0339 24710308 PMC4012961

[15] Ferroni P Guadagni F Riondino S Evaluation of mean platelet volume as a predictive marker for cancer-associated venous thromboembolism during chemotherapy Haematologica 2014 99 10 1638 1644 10.3324/haematol.2014.109470 25085351 PMC4181262

[16] Ferroni P Palmirotta R Riondino S VEGF gene promoter polymorphisms and risk of VTE in chemotherapy-treated cancer patients Thromb Haemost 2016 115 01 143 151 10.1160/TH15-03-0259 26336029

[17] Ferroni P Riondino S Formica V Venous thromboembolism risk prediction in ambulatory cancer patients: clinical significance of neutrophil/lymphocyte ratio and platelet/lymphocyte ratio Int J Cancer 2015 136 05 1234 1240 10.1002/ijc.29076 25042739

[18] Roselli M Ferroni P Riondino S Impact of chemotherapy on activated protein C-dependent thrombin generation–association with VTE occurrence Int J Cancer 2013 133 05 1253 1258 10.1002/ijc.28104 23404208

[19] Khorana A A Francis C W Culakova E Lyman G H Risk factors for chemotherapy-associated venous thromboembolism in a prospective observational study Cancer 2005 104 12 2822 2829 10.1002/cncr.21496 16284987

[20] van Es N Di Nisio M Cesarman G Comparison of risk prediction scores for venous thromboembolism in cancer patients: a prospective cohort study Haematologica 2017 102 09 1494 1501 10.3324/haematol.2017.169060 28550192 PMC5685240

[21] van Es N Hisada Y Di Nisio M Extracellular vesicles exposing tissue factor for the prediction of venous thromboembolism in patients with cancer: A prospective cohort study Thromb Res 2018 166 54 59 10.1016/j.thromres.2018.04.009 29656167

[22] Di Nisio M van Es N Rotunno L Long-term performance of risk scores for venous thromboembolism in ambulatory cancer patients J Thromb Thrombolysis 2019 48 01 125 133 10.1007/s11239-019-01845-6 30919253

[23] Abdel-Razeq H Mansour A Abdulelah H Thromboembolic events in cancer patients on active treatment with cisplatin-based chemotherapy: another look! Thromb J 2018 16 2 10.1186/s12959-018-0161-9 29507532 PMC5831696

[24] Tafur A J Dale G Cherry M Prospective evaluation of protein C and factor VIII in prediction of cancer-associated thrombosis Thromb Res 2015 136 06 1120 1125 10.1016/j.thromres.2015.10.004 26475410 PMC4679511

[25] Arpaia G Carpenedo M Verga M D-dimer before chemotherapy might predict venous thromboembolism Blood Coagul Fibrinolysis 2009 20 03 170 175 10.1097/MBC.0b013e32831bc2de 19276795

[26] Vergati M Della-Morte D Ferroni P Increased risk of chemotherapy-associated venous thromboembolism in elderly patients with cancer Rejuvenation Res 2013 16 03 224 231 10.1089/rej.2013.1409 23521603

[27] COMPASS–CAT Working Group Gerotziafas G T Taher A Abdel-Razeq H A Predictive Score for Thrombosis Associated with Breast, Colorectal, Lung, or Ovarian Cancer: The Prospective COMPASS-Cancer-Associated Thrombosis Study Oncologist 2017 22 10 1222 1231 10.1634/theoncologist.2016-0414 28550032 PMC5634762

[28] Bosch F TM Mulder F I Kamphuisen P W Primary thromboprophylaxis in ambulatory cancer patients with a high Khorana score: a systematic review and meta-analysis Blood Adv 2020 4 20 5215 5225 10.1182/bloodadvances.2020003115 33104795 PMC7594395

[29] Doggen C J Thromboprophylaxis in cancer outpatients Blood 2008 111 10 4833 10.1182/blood-2008-02-137760 18467600

[30] Verso M Agnelli G Barni S Gasparini G LaBianca R A modified Khorana risk assessment score for venous thromboembolism in cancer patients receiving chemotherapy: the Protecht score Intern Emerg Med 2012 7 03 291 292 10.1007/s11739-012-0784-y 22547369

[31] Blom J W Doggen C J Osanto S Rosendaal F R Malignancies, prothrombotic mutations, and the risk of venous thrombosis JAMA 2005 293 06 715 722 10.1001/jama.293.6.715 15701913

[32] Haddad T C Greeno E W Chemotherapy-induced thrombosis Thromb Res 2006 118 05 555 568 10.1016/j.thromres.2005.10.015 16388837

[33] Elliott M A Wolf R C Hook C C Thromboembolism in adults with acute lymphoblastic leukemia during induction with L-asparaginase-containing multi-agent regimens: incidence, risk factors, and possible role of antithrombin Leuk Lymphoma 2004 45 08 1545 1549 10.1080/10428190410001693588 15370205

[34] Dickmann B Ahlbrecht J Ay C Regional lymph node metastases are a strong risk factor for venous thromboembolism: results from the Vienna Cancer and Thrombosis Study Haematologica 2013 98 08 1309 1314 10.3324/haematol.2012.073338 23585523 PMC3729913

[35] Alahmari A K Almalki Z S Alahmari A K Guo J J Thromboembolic Events Associated with Bevacizumab plus Chemotherapy for Patients with Colorectal Cancer: A Meta-Analysis of Randomized Controlled Trials Am Health Drug Benefits 2016 9 04 221 232 27688834 PMC5004819

[36] Li L J Chen D F Wu G F Incidence and risk of thromboembolism associated with bevacizumab in patients with non-small cell lung carcinoma J Thorac Dis 2018 10 08 5010 5022 10.21037/jtd.2018.07.09 30233875 PMC6129907

[37] Numico G Garrone O Dongiovanni V Prospective evaluation of major vascular events in patients with nonsmall cell lung carcinoma treated with cisplatin and gemcitabine Cancer 2005 103 05 994 999 10.1002/cncr.20893 15666321

[38] Dasanu C A Gemcitabine: vascular toxicity and prothrombotic potential Expert Opin Drug Saf 2008 7 06 703 716 10.1517/14740330802374262 18983217

