# Supplementary material for: Prognostic Factors for Venous Thromboembolism in Patients with Solid Tumours on Systemic Therapy: A Systematic Review
Source: TH Open. 2021 Sep 10;5(3):e461–9. doi: 10.1055/a-1642-4572 (PMC8483896; doi:10.1055/a-1642-4572)
Supplement: Supplementary file 1 — Supplementary Material [file 10-1055-a-1642-4572-s210033.pdf]

Supplementary Table S1 OVID Medline Epub Ahead of Print, In-Process & Other Non-Indexed Citations, Ovid MEDLINE ® Daily and Ovid MEDLINE ® 1946 to August 2019 Search Strategy

1. Venous thromboembolism/
2. exp Venous Thrombosis/
3. exp Pulmonary Embolism/
4. dvt.mp.
5. pulmonary embolism.ti,ab.
6. ((ven* or vein*) adj2 thromb*).ti,ab.
7. or/1–6
8. exp Angiogenesis Inhibitors/
9. exp Anticarcinogenic Agents/
10. exp Antimetabolites, antineoplastic/
11. exp Antimitotic Agents/
12. exp Antineoplastic agents, alkylating/
13. exp Antineoplastic agents, immunological/
14. exp Topoisomerase inhibitors/
15. immunotherap*.ti,ab
16. systemic therapy.ti,ab.
17. chemo*.ti,ab.
18. antineoplastic.ti,ab.
19. targeted therap*.ti,ab.
20. targetted therap*.ti,ab.
21. biologic* agent*.ti,ab.
22. antitumor.ti,ab.
23. antitumor.ti,ab.
24. or/8–23
25. exp Neoplasms/
26. cancer*.ti,ab.
27. malig*.ti,ab.
28. neoplas*.ti,ab.
29. oncol*.ti,ab.
30. tumor*.ti,ab.
31. tumor*.ti,ab
32. or/25–31
33. exp Risk Assessment/
34. Incidence/
35. Incidence Studies/
36. Risk Factors/
37. associat*.ti,ab.
38. validat*.ti,ab.
39. incidence*.ti,ab.
40. predict*.ti,ab.
41. risk factor*.ti,ab.
42. prognos*.ti,ab.
43. or/33–42
44. 7 and 24 and 32 and 43
45. limit 38 to ("review" or "scientific integrity review" or "systematic review" or systematic reviews as topic)
46. exp Animals/ not exp Humans/
47. 44 not 45
48. 47 not 46

Supplementary Table S2 Embase 1974 to August 2019 Search Strategy

1. exp Venous thromboembolism/
2. exp Vein Thrombosis/
3. exp Lung Embolism/
4. dvt.ti,ab.
5. pulmonary embolism.ti,ab.
6. ((ven* or vein*) adj2 thromb*).ti,ab.
7. or/1–6
8. Antineoplastic Agent/
9. immunotherap*.ti,ab
10. systemic therapy.ti,ab.
11. chemo*.ti,ab.
12. antineoplastic.ti,ab.
13. targeted therap*.ti,ab.
14. targetted therap*.ti,ab.
15. biologic* agent*.ti,ab.
16. antitumor.ti,ab.
17. antitumor.ti,ab.
18. or/8–17
19. exp Malignant Neoplasm/
20. cancer*.ti,ab.
21. malig*.ti,ab.
22. neoplas*.ti,ab.
23. oncol*.ti,ab.
24. tumor*.ti,ab.
25. tumor*.ti,ab
26. or/19–25
27. exp Risk Assessment/
28. Incidence/
29. Risk Factor/
30. validat*.ti,ab.
31. incidence*.ti,ab.
32. predict*.ti,ab.
33. risk factor*.ti,ab.
34. prognos*.ti,ab.
35. or/27–34
36. 7 and 18 and 26 and 35
37. limit 36 to ("review")
38. exp Animal/ not exp Human/
39. 36 not 37
40. 39 not 38

 © 2021. The Author(s).

Supplementary Table S3 Cochrane Controlled Register of Trials (CENTRAL) Database Conception to August 2019 Search Strategy

1. Venous thromboembolism/
2. exp Venous Thrombosis/
3. exp Pulmonary Embolism/
4. dvt.mp.
5. pulmonary embolism.ti,ab.
6. ((ven* or vein*) adj2 thromb*).ti,ab.
**7. or/1–6**
8. exp Angiogenesis Inhibitors/
9. exp Anticarcinogenic Agents/
10. exp Antimetabolites, antineoplastic/
11. exp Antimitotic Agents/
12. exp Antineoplastic agents, alkylating/
13. exp Antineoplastic agents, immunological/
14. exp Topoisomerase inhibitors/
15. ssociateapy*.ti,ab
16. systemic therapy.ti,ab.
17. chemo*.ti,ab.
18. antineoplastic.ti,ab.
19. targeted therap*.ti,ab.
20. ssociat therap*.ti,ab.
21. biologic* agent*.ti,ab.
22. antitumor.ti,ab.
23. antitumor.ti,ab.
**24. or/8–23**
25. exp Neoplasms/
26. cancer*.ti,ab.
27. malig*.ti,ab.
28. neoplas*.ti,ab.
29. oncol*.ti,ab.
30. tumor*.ti,ab.
31. tumor*.ti,ab
**32. or/25–31**
33. exp Risk Assessment/
34. Incidence/
35. Risk Factors/
36. associate*.ti,ab.
37. validat*.ti,ab.
38. incidence*.ti,ab.
39. predict*.ti,ab.
40. risk factor*.ti,ab.
41. prognos*.ti,ab.
**42. or/33–41**
**43. 7 and 24 and 32 and 42**

 © 2021. The Author(s).

Supplementary Table S4 Risk of bias assessment using the QUIPS Tool

Author and year	Study participation	Study attrition	Prognostic factor measurement	Outcome measurement	Study confounding	Statistical analysis and reporting	Overall study quality
Abdel-Razeq 2018	high risk	low risk	moderate risk	low risk	low risk	low risk	high risk
Arpaia 2009	low risk	low risk	low risk	low risk	high risk	moderate risk	high risk
Di Nisio 2019	high risk	high risk	moderate risk	low risk	high risk	low risk	high risk
Ferroni (GFR) 2014*	low risk	moderate risk	moderate risk	low risk	moderate risk	high risk	high risk
Ferroni (MPV) 2014*	high risk	high risk	moderate risk	low risk	moderate risk	high risk	high risk
Ferroni 2015*	high risk	low risk	moderate risk	low risk	low risk	moderate risk	high risk
Ferroni 2016	low risk	low risk	moderate risk	low risk	high risk	moderate risk	high risk
Gerotziafas 2017	low risk	moderate risk	low risk	low risk	moderate risk	low risk	moderate risk
Khorana 2005**	high risk	moderate risk	low risk	low risk	low risk	high risk	high risk
Khorana 2008**	high risk	moderate risk	moderate risk	low risk	low risk	moderate risk	high risk
Roselli 2013*	low risk	high risk	moderate risk	low risk	high risk	high risk	high risk
Tafur 2015	high risk	high risk	low risk	low risk	moderate risk	high risk	high risk
van Es 2017***	high risk	high risk	low risk	low risk	moderate risk	high risk	high risk
van Es 2018***	high risk	high risk	low risk	low risk	high risk	high risk	high risk
Vergati 2013*	low risk	low risk	low risk	low risk	moderate risk	high risk	high risk

*Study conducted by same research group
**Study conducted by same research group
***Study conducted by same research group
The domain with the highest risk of bias was used to determine the overall study quality.

 © 2021. The Author(s).

Supplementary Table S5 Result of multivariable analysis of prognostic factors for VTE

Prognostic Factor	Studies	Sample size	Definition	Measure of association	Risk estimate[1] (95% CI)	Pos/neg association[2]
**Patient Factors**						
Age	Vergati 2013	486	>70 vs ≤70 years	HR	**2.43 (1.16–5.09)**	Pos
	Arpaia 2009	124	ND	HR	1.04 (0.9–1.09)	Pos
	van Es 2018***	684	per 1 year increase	SHR	1.00 (0.96–1.03)	N/A
	Roselli 2013*	505	>65 vs ≤65 years	HR	0.99 (0.49–2.02)	Neg
	Ferroni 2014 (MPV)*	589	ND	HR	1.00 (0.97–1.02)	N/A
	Ferroni 2014 (GFR)*	322	>65 vs ≤65 years	OR	0.97 (0.38–2.46)	Pos
	Ferroni 2015*	380	>65 vs ≤65 years	HR	0.78 (0.35–1.74)	Neg
	Ferroni 2016*	297	>65 vs ≤65 years	HR	1.97 (0.82–4.71)	Pos
Gender	Abdel-Razeq 2018	1677	vs male	OR	**1.732 (1.152–2.605)**	Pos
	Vergati 2013	486	vs male	HR	0.60 (0.28–1.29)	Neg
	van Es 2018***	648	vs male	SHR	1.05 (2.13–0.53)	Pos
	Roselli 2013*	505	ND	HR	0.79 (0.39–1.59)	Neg
	Ferroni 2014 (MPV)*	589	ND	HR	1.15 (0.57–2.33)	Pos
	Ferroni 2014 (GFR)*	322	ND	OR	1.26 (0.48–3.36)	Pos
	Ferroni 2015*	380	vs male	HR	1.38 (0.57–3.35)	Pos
	Ferroni 2016*	297	vs male	HR	1.40 (0.57–3.43)	Pos
BMI	Di Nisio 2019	776	>35 vs ≤35	SHR	1.6 (0.50–5.4)[a]	Pos
				SHR	1.8 (0.55–5.9)[b]	Pos
	Khorana 2008**	2701	>35 vs ≤35	OR	**2.5 (1.3–4.7)**	Pos
	Ferroni 2015*	380	>35 vs ≤35	HR	0.46 (0.03–7.18)	Neg
	Roselli 2013*	505	ND	HR	1.04 (0.97–1.11)	Pos
ECOG Status	Di Nisio 2019	776	≥2 vs <2 points	SHR	1.5 (0.46–4.6)[c]	Pos
	van Es 2017***	876	≥2 vs <2 points	SHR	1.2 (0.5–2.9)[c]	Pos
	Vergati 2013	486	2 vs <2 points	HR	**6.63 (3.13–14.0)**	Pos
	Ferroni 2014 (MPV)*	589		HR	**2.25 (1.18–4.27)**	Pos
	Roselli 2013*	505		HR	**4.26 (2.15–8.43)**	Pos
	Ferroni 2014 (GFR)*	322		OR	1.72 (0.65–4.52)	Pos
	Ferroni 2015*	380		HR	**2.57 (1.34–4.93)**	Pos

(*Continued*)

Supplementary Table S5 (*Continued*)

Prognostic Factor	Studies	Sample size	Definition	Measure of association	Risk estimate[1] (95% CI)	Pos/neg association[2]
	Ferroni 2016*	297		HR	**4.13 (1.84–9.27)**	Pos
Khorana Score	Abdel-Razeq 2018	1677	≥3 vs <3 points	OR	1.387 (0.842–2.285)	Pos
	Di Nisio 2019	776	≥2 vs <2 points	SHR	1.8 (0.95–3.4)[d]	Pos
	Vergati 2013	486	ND	HR	1.34 (0.7–2.58)	Pos
	van Es 2018***	648	per 1 point increase	SHR	1.008 (0.768–1.32)	Pos
	Ferroni 2016*	297	ND	HR	**4.13 (1.84–9.27)**	Pos
	Ferroni 2014 (MPV)*	589	ND	HR	1.15 (0.62–2.15)	Pos
	Ferroni 2014 (GFR)*	322	ND	OR	2.42 (0.91–6.48)	Pos
Previous VTE	Di Nisio 2019	776	Any VTE that occurred 6+ months before enrollment	SHR	**17 (5.2–52)**[d]	Pos
Vascular/lymphatic macroscopic compression	Di Nisio 2019	776	Vascular/lymphatic macroscopic compression by tumor, detected by venous compression ultrasound (B-mode imaging)	SHR	1.7 (0.60–5.0)[d]	Pos
Cardiovascular comorbidities	Gerotziafas 2017	1023	At least 2 of: personal history of peripheral artery disease, ischemic stroke, coronary artery disease, hypertension, hyperlipidemia, diabetes, obesity	OR	**5.18 (1.10–13.40)**	Pos
**Tumour Factors**						
Tumor site (High risk vs low risk)	Di Nisio 2019	776	High Risk (Lung, Lymphoma, Gynecologic, Genitourinary Except Prostate) vs Low Risk (breast, colorectal, head-neck)	SHR	1.9 (0.71–4.9)[a]	Pos
				SHR	2.2 (0.83–6.0)[b]	Pos
				SHR	1.8 (0.68–4.7)[c]	Pos
	Khorana 2008**	2701		OR	1.5 (0.9–2.7)	Pos
	van Es 2017***	876		SHR	1.10 (0.59–2.10)[a]	Pos
				SHR	1.00 (0.53–1.80)[e]	N/A
				SHR	0.66 (0.33–1.3)[b]	Neg
				SHR	1.1 (0.58–2)[c]	Pos

Supplementary Table S5 (*Continued*)

Prognostic Factor	Studies	Sample size	Definition	Measure of association	Risk estimate[1] (95% CI)	Pos/neg association[2]
Tumor site (Very high risk vs low risk)	Di Nisio 2019	776	Very High Risk (Stomach, Pancreas) vs Low Risk (breast, colorectal, head-neck)	SHR	1.8 (0.85–3.9)[a]	Pos
				SHR	1.7 (0.72–3.8)[b]	Pos
				SHR	1.8 (0.85–3.9)[c]	Pos
	Khorana 2008**	2701		OR	**4.3 (1.2–15.6)**	Pos
	Ferroni 2015*	380		HR	1.32 (0.63–2.80)	Pos
	Khorana 2005**	3003		OR	**3.88 (1.43–10.05)**	Pos
	van Es 2017***	876		SHR	1.40 (0.72–2.90)[a]	Pos
				SHR	1.50 (0.72–2.90)[e]	Pos
				SHR	1.10 (0.54–2.20)[b]	Pos
				SHR	1.50 (0.72–2.90)[c]	Pos
Tumor site (Other)	Abdel-Razeq 2018	1677	Gastric vs others	OR	**3.377 (1.759–6.483)**	Pos
	Vergati 2013	486	ND	HR	0.97 (0.84–1.10)	Neg
	Ferroni 2014 (MPV)*	589	ND	HR	1.07 (0.76–1.51)	Pos
	Ferroni 2014 (GFR)*	322	ND	OR	1.63 (0.94–2.82)	Pos
Tumor stage	Di Nisio 2019	776	Metastatic vs Non-metastatic	SHR	1.1 (0.52–2.30)[d]	Pos
	van Es 2018***	648		SHR	1.26 (0.61–2.61)	Pos
	Vergati 2013	486	Non-metastatic vs Metastatic	HR	0.88 (0.31–2.48)	Neg
	Roselli 2013*	505	Metastatic vs Non-metastatic	HR	1.87 (0.76–4.65)	Pos
	Arpaia 2009	124		HR	1.25 (0.35–4.37)	Pos
	Abdel-Razeq 2018	1677		OR	**1.67 (1.05–2.63)**	Pos
	Ferroni 2015*	380		HR	1.01 (0.45–2.25)	Pos
	Ferroni 2014 (GFR)*	322		OR	1.32 (0.49–3.55)	Pos
	Ferroni 2014 (MPV)*	589	ND	HR	1.15 (0.93–1.41)	Pos
	Ferroni 2016*	297	Relapsing/metastatic	HR	1.41 (0.53–3.75)	Pos
	Gerotziafas 2017	1023	Advanced stage or metastatic	OR	1.93 (0.92–2.64)	Pos
Time since cancer diagnosis	Gerotziafas 2017	1023	≤6 months since diagnosis	OR	**4.10 (2.10–7.98)**	Pos

(*Continued*)

 © 2021. The Author(s).

Supplementary Table S5 (*Continued*)

Prognostic Factor	Studies	Sample size	Definition	Measure of association	Risk estimate[1] (95% CI)	Pos/neg association[2]
**Biomarkers**						
Prechemotherapy hemoglobin	Di Nisio 2019	776	Prechemotherapy hemoglobin <100 g/L or use of erythropoietin stimulating agents	SHR	1.5 (0.58–4.1)[a]	Pos
				SHR	1.7 (0.63–4.6)[b]	Pos
				SHR	1.6 (0.59–4.1)[c]	Pos
	Khorana 2005**	3003		OR	**1.83 (1.07–3.14)**	Pos
	van Es 2017***	876		SHR	0.76 (0.32–1.80)[a]	Neg
				SHR	0.64 (0.27–1.60)[e]	Neg
				SHR	0.70 (0.28–1.70)[b]	Neg
				SHR	0.76 (0.31–1.80)[c]	Neg
	Khorana 2008**	876		OR	**2.4 (1.4–4.2)**	Pos
	Ferroni 2015*	380	Haemoglobin <100 g L^-11	HR	1.54 (0.43–5.50)	Pos
Prechemotherapy white blood cell count	Di Nisio 2019	776	Prechemotherapy white blood cell count >11 × 10^9/L	SHR	1.7 (0.75–3.6)[a]	Pos
				SHR	1.81 (0.80–4.1)[b]	Pos
				SHR	1.7 (0.76–3.6)[c]	Pos
	Khorana 2008**	2701		OR	**2.2 (1.2–4)**	Pos
	Ferroni 2015*	380		HR	0.79 (0.23–2.65)	Neg
	van Es 2017***	876		SHR	0.87 (0.42–1.80)[a]	Neg
				SHR	0.82 (0.39–1.80)[e]	Neg
				SHR	0.77 (0.36–1.7)[b]	Neg
				SHR	0.86 (0.41–1.8)[c]	Neg
Prechemotherapy platelet count	Di Nisio 2019	776	≥ 350 × 10^9/L	SHR	0.97 (0.45–2.1)[a]	Neg
				SHR	0.82 (0.38–1.8)[b]	Neg
				SHR	0.98 (0.46–2.1)[c]	Neg
	Khorana 2008**	2701		OR	**1.8 (1.1–3.2)**	Pos
	Gerotziafas 2017	1023		OR	**2.53 (1.35–4.74)**	Pos
	van Es 2017***	876		SHR	1.40 (0.80–2.40)[a]	Pos
				SHR	1.30 (0.71–2.20)[e]	Pos
				SHR	1.30 (0.74–2.30)[b]	Pos
				SHR	1.40 (0.80–2.40)[c]	Pos
	Khorana 2005**	3003		OR	**2.81 (1.63–4.93)**	Pos
	Ferroni 2015*	380	>350 × 10^9/L	HR	0.18 (0.03–1.02)	Neg
	Ferroni 2014 (MPV)*	589	≤7.3 fL or >7.3 fL	HR	**2.32 (1.03–5.23)**	Pos

 © 2021. The Author(s).

Supplementary Table S5 (*Continued*)

Prognostic Factor	Studies	Sample size	Definition	Measure of association	Risk estimate[1] (95% CI)	Pos/neg association[2]
Low protein C activity (%)	Tafur 2015	241	Dichotomized as ≤ 118% (lower quartile) and >118%	HR	**2.5 (1.1–5.5)**	Pos
High factor VIII activity (%)	Tafur 2015	241	Dichotomized as ≤ 261% and >261%	HR	**3.0 (1.1–8.0)**	Pos
ThromboPath change (%)	Roselli 2013*	505	Decreasing (-6% cutoff) or not	HR	**2.39 (1.20–4.78)**	Pos
D-dimer levels	van Es 2018***	648	Per 1 ug/L [log transformed] increase	SHR	**1.91 (1.40–2.62)**	Pos
	Arpaia 2009	124	>650 ng/mL	HR	**4.04 (1.22–13.3)**	Pos
	van Es 2017***	876	>1.44 g/L	SHR	**2.40 (1.30–4.40)**[e]	Pos
	Vergati 2013	486	Negative vs positive (cutoff of 280 ng/mL)	HR	3.11 (0.83–11.7)	Pos
Fibrin generation test	van Es 2018***	648	High versus low activity	SHR	1.94 (0.99–3.80)	Pos
eGFR	Ferroni 2014 (GFR)*	322	eGFR categorized as normal or impaired based on the cutoff of normal being >90 mL/min per 1.73 m	OR	**3.44 (1.27–9.34)**	Pos
Platelet/lymphocyte ratio	Ferroni 2015*	380	Platelet/lymphocyte ratio >260	HR	**2.72 (1.10–6.73)**	Pos
Neutrophil/lymphocyte ratio	Ferroni 2015*	380	Neutrophil/lymphocyte ratio >3	HR	1.37 (0.52–3.57)	Pos
**Treatment Factors**						
Hospitalization	Gerotziafas 2017	1023	Hospitalization for any non-surgical reason occurring within the last 3 months before assessment	OR	**5.41 (2.90–10.08)**	Pos
Adjuvant chemotherapy	Arpaia 2009	124	vs first-line chemotherapy	HR	0.69 (0.07–6.93)	Neg
Central venous catheter	Abdel-Razeq 2018	1677	vs no catheter	OR	1.098 (0.603–1.999)	Pos
	Gerotziafas 2017	1023	vs no catheter	OR	**3.24 (1.56–6.72)**	Pos
Cytotoxic Chemotherapy						
Gemcitabine	Di Nisio 2019	776	vs other treatment	SHR	0.70 (0.34–1.5)[b]	Neg
	van Es 2017***	876		SHR	**3.70 (1.80–7.60)**[b]	Pos
	Vergati 2013	486		HR	0.42 (0.11–1.56)	Neg
	Ferroni 2014 (MPV)*	589		HR	0.66 (0.23–1.90)	Neg
	Ferroni 2015*	380		HR	0.45 (0.16–1.32)	Neg
	Ferroni 2016*	297		HR	1.78 (0.23–13.9)	Pos

(*Continued*)

Supplementary Table S5 (*Continued*)

Prognostic Factor	Studies	Sample size	Definition	Measure of association	Risk estimate[1] (95% CI)	Pos/neg association[2]
	Ferroni 2014 (GFR)*	322		OR	0.51 (0.14–1.82)	Neg
Platinum	Vergati 2013	486	vs other treatment	HR	**2.51 (1.08–5.83)**	Pos
	Ferroni 2014 (MPV)*	589		HR	1.25 (0.53–2.95)	Pos
	Di Nisio 2019	776		SHR	**2.5 (1.1–6.1)**[b]	Pos
	van Es 2017***	876		SHR	**2.8 (1.4–5.6)**[b]	Pos
	Roselli 2013*	505		HR	**2.19 (1.03–4.68)**	Pos
	Ferroni 2016*	297		HR	**4.86 (1.27–18.60)**	Pos
	Ferroni 2015*	380		HR	0.86 (0.35–2.13)	Neg
Fluoropyrimidine	Ferroni 2016*	297	vs other treatment	HR	3.71 (0.61–22.36)	Pos
	Roselli 2013*	505		HR	1.57 (0.69–3.55)	Pos
	Ferroni 2014 (MPV)*	589		HR	0.89 (0.35–2.25)	Neg
	Ferroni 2014 (GFR)*	322		OR	2.27 (0.36–14.30)	Pos
	Ferroni 2015*	380		HR	0.54 (0.16–1.85)	Neg
Irinotecan	Ferroni 2016*	297	vs other treatment	HR	1.44 (0.36–5.70)	Pos
	Ferroni 2014 (MPV)*	589		HR	0.85 (0.25–2.897)	Neg
	Ferroni 2015*	380		HR	0.85 (0.16–4.45)	Neg
Anthracycline	Ferroni 2016*	297	vs other treatment	HR	0.18 (0.02–1.87)	Neg
	Ferroni 2014 (MPV)*	589		HR	0.40 (0.08–1.95)	Neg
	Ferroni 2014 (GFR)*	322		OR	0.33 (0.04–1.50)	Neg
	Gerotziafas 2017	1023		OR	**2.33 (1.02–5.33)**	Pos
	Ferroni 2015*	380		HR	1.33 (0.31–5.68)	Pos
Docetaxel	Ferroni 2016*	297	vs other treatment	HR	**7.47 (1.18–47.4)**	Pos
	Ferroni 2014 (MPV)*	589		HR	0.92 (0.30–2.88)	Neg
	Ferroni 2014 (GFR)*	322		OR	0.25 (0.04–1.50)	Neg
	Ferroni 2015*	380		HR	0.71 (0.21–2.42)	Neg
Pemetrexed	Ferroni 2014 (MPV)*	589	vs other treatment	HR	0.75 (0.19–2.94)	Neg
	Ferroni 2014 (GFR)*	322		OR	0.11 (0.01–1.14)	Neg
	Ferroni 2015*	380		HR	0.43 (0.11–1.76)	Neg

 © 2021. The Author(s).

Supplementary Table S5 (*Continued*)

Prognostic Factor	Studies	Sample size	Definition	Measure of association	Risk estimate[1] (95% CI)	Pos/neg association[2]
Biological Therapy						
Bevacizumab	Ferroni 2016*	297	vs other treatment	HR	**3.56 (1.12–11.40)**	Pos
	Roselli 2013*	505		HR	1.21 (0.43–3.40)	Pos
	Ferroni 2014 (MPV)*	589		HR	2.59 (0.84–1.15)	Pos
	Vergati 2013	486		HR	2.49 (0.84–7.32)	Pos
	Ferroni 2014 (GFR)*	322		OR	1.00 (0.10–9.87)	N/A
	Ferroni 2015*	380		HR	**4.54 (1.27–16.2)**	Pos
Herceptin	Ferroni 2014 (MPV)*	589	vs other treatment	HR	0.96 (0.11–8.11)	Neg
	Ferroni 2015*	380		HR	0 (No events)	N/A
	Ferroni 2014 (GFR)*	322		OR	0 (No events)	N/A
Anti-tyrosine kinase inhibitors	Ferroni 2014 (MPV)*	589	vs other treatment	HR	1.23 (0.13–11.5)	Pos
	Vergati 2013	486		HR	1.75 (0.21–14.7)	Pos
	Ferroni 2015*	380		HR	6.29 (0.59–67.2)	Pos
Other						
Endocrine/anti-hormonal therapy	Ferroni 2014 (MPV)*	589	vs other treatment	HR	0.78 (0.09–6.80)	Neg
	Gerotziafas 2017	1023	vs other treatment in women with breast cancer	OR	**6.40 (3.16–12.96)**	Pos
Supportive drug use during chemotherapy						
Erythropoietin stimulating agents	Di Nisio 2019	776	vs none	SHR	1.5 (0.58–4.1)[a]	Pos
				SHR	1.7 (0.63–4.6)[b]	Pos
				SHR	1.6 (0.59–4.1)[c]	Pos
	Ferroni 2015*	380		HR	1.49 (0.34–6.66)	Pos
	Roselli 2013*	505		HR	1.33 (0.32–5.55)	Pos
	van Es 2017***	876		SHR	0.76 (0.32–1.80)[a]	Neg
				SHR	0.64 (0.27–1.60)[e]	Neg
				SHR	0.70 (0.28–1.70)[b]	Neg
				SHR	0.76 (0.31–1.80)[c]	Neg
Prophylactic myeloid growth factors	Roselli 2013*	505	vs none	HR	1.25 (0.31–5.08)	Pos
	Ferroni 2014 (MPV)*	589		HR	0.97 (0.76–1.23)	Neg
	Vergati 2013	486		HR	0.60 (0.08–4.61)	Neg
	Ferroni 2014 (GFR)*	322		OR	1.18 (0.19–7.37)	Pos
	Ferroni 2015*	380		HR	1.00 (0.22–4.73)	Pos

(*Continued*)

Supplementary Table S5 (*Continued*)

Prognostic Factor	Studies	Sample size	Definition	Measure of association	Risk estimate[1] (95% CI)	Pos/neg association[2]
Corticosteroids	Roselli 2013*	505	vs none	HR	1.18 (0.52–2.65)	Pos
	Ferroni 2014 (MPV)*	589		HR	0.98 (0.84–1.15)	Neg
	Vergati 2013	486		HR	1.84 (0.86–3.94)	Pos
	Ferroni 2014 (GFR)*	322		OR	1.50 (0.48–4.66)	Pos
	Ferroni 2015*	380		HR	1.16 (0.46–2.88)	Pos
**Genetic factors**						
VEGFA-1190G/A A/A polymorphism	Ferroni 2016*	297	vs none	HR	1.24 (0.54–2.88)	Pos
VEGFA-1154G/A A/A polymorphism	Ferroni 2016*	297	vs none	HR	**0.21 (0.07–0.58)**	Neg
VEGFA-634G/C C/C polymorphism	Ferroni 2016*	297	vs none	HR	0.82 (0.38–1.77)	Neg

Abbreviations: ECOG, Eastern Cooperative Oncology Group; ND, Not Defined (in the original study).
*Study conducted by same research group.
**Study conducted by same research group.
***Study conducted by same research group.
[1]Statistically significant results are indicated in bold text.
[2]Positive association refers to a point estimate greater than 1 and a negative association refers to a point estimate less than 1.
[a]Adjusted by Khorana score variables (Pancreatic or gastric cancer; lung, ovarian, urogenital; prechemotherapy hemoglobin; prechemotherapy white blood cell count; prechemotherapy platelet count; Body mass index >35).
[b]Adjusted by PROTECHT score variables (Pancreatic or gastric cancer; lung, ovarian, urogenital; prechemotherapy hemoglobin; prechemotherapy white blood cell count; prechemotherapy platelet count; Body mass index >35; gemcitabine chemotherapy; platinum-based chemotherapy).
[c]Adjusted by CONKO score variables (Pancreatic or gastric cancer; lung, ovarian, urogenital; prechemotherapy hemoglobin; prechemotherapy white blood cell count; prechemotherapy platelet count; ECOG performance status ≥2).
[d]Adjusted by ONKOTEV score variables (Khorana score ≥2; metastasis; previous venous thromboembolism; vascular/lymphatic macroscopic compression.
[e]Adjusted by Vienna CATS score variables (Pancreatic or gastric cancer; lung, ovarian, urogenital; prechemotherapy hemoglobin; prechemotherapy white blood cell count; prechemotherapy platelet count; Body mass index >35; D-dimer; soluble P-selectin.

 © 2021. The Author(s).
